# Multi-Strain Probiotics: Synergy among Isolates Enhances Biological Activities

**DOI:** 10.3390/biology10040322

**Published:** 2021-04-13

**Authors:** Iliya D. Kwoji, Olayinka A. Aiyegoro, Moses Okpeku, Matthew A. Adeleke

**Affiliations:** 1 Discipline of Genetics, School of Life Sciences, Westville Campus, University of KwaZulu-Natal, Durban 4000, South Africa; 219094534@stu.ukzn.ac.za (I.D.K.); okpekum@ukzn.ac.za (M.O.); 2 Gastrointestinal Microbiology and Biotechnology Unit, Agricultural Research Council-Animal Production, Irene 0062, South Africa; aiyegoroo@arc.agric.za; 3 Unit for Environmental Sciences and Management, North-West University, Potchefstroom 2520, South Africa

**Keywords:** antibiotics, probiotics, cell-cell communication, synergy, antagonism

## Abstract

**Simple Summary:**

Multi-strain probiotics are composed of more than one species or strains of bacteria and sometimes, including some fungal species with benefits to human and animals’ health. The mechanisms by which multi-strain probiotics exert their effects include cell–cell communications, interactions with the host tissues, and modulation of the immune systems. Multi-strain probiotics applications include alleviation of disease conditions, inhibition of pathogens, and restoration of the gastrointestinal microbiome. Despite all these benefits, the potential of using multi-strain probiotics is still not fully explored.

**Abstract:**

The use of probiotics for health benefits is becoming popular because of the quest for safer products with protective and therapeutic effects against diseases and infectious agents. The emergence and spread of antimicrobial resistance among pathogens had prompted restrictions over the non-therapeutic use of antibiotics for prophylaxis and growth promotion, especially in animal husbandry. While single-strain probiotics are beneficial to health, multi-strain probiotics might be more helpful because of synergy and additive effects among the individual isolates. This article documents the mechanisms by which multi-strain probiotics exert their effects in managing infectious and non-infectious diseases, inhibiting antibiotic-resistant pathogens and health improvement. The administration of multi-strain probiotics was revealed to effectively alleviate bowel tract conditions, such as irritable bowel syndrome, inhibition of pathogens and modulation of the immune system and gut microbiota. Finally, while most of the current research focuses on comparing the effects of multi-strain and single-strain probiotics, there is a dearth of information on the molecular mechanisms of synergy among multi-strain probiotics isolates. This forms a basis for future research in the development of multi-strain probiotics for enhanced health benefits.

## 1. Introduction

The Food and Agriculture Organization/World Health Organization (FAO/WHO) working committee on probiotics defined probiotics as “live microorganisms which when administered in adequate amounts confer health benefits on the host” [1]. These microbial dietary preparations could exert valuable functions on man and animals’ physiology through modulation of systemic and mucosal immunity and restore the dietary and microbial balances within the gastrointestinal system [2,3]. The use of probiotics is increasing due to consideration as a suitable option following restrictions on antibiotics as growth promoters in the livestock industries by many countries [4]. Probiotics have gained several applications such as inhibition of pathogen [5], improvement of animals health and performance, and pond quality in aquaculture [6,7]. Previous studies revealed that some probiotics might affect health indexes, microbiome structure, and inhibit pathogenic microbes’ population within the gut [8,9,10]. The strains of microorganisms used as probiotics include members of the genus *Bacillus*, *Enterococcus*, *Lactobacillus*, *Pedicoccus*, *Streptococcus*, *Propionibacterium, Bifidobacterium, Saccharomyces, Debaryomyces, Micrococcus*, and *Photobacterium* among others [11]. Recently, different studies are also proposing the use of some commensal clostridial species as probiotics due to their spore formation [12] and stimulation of T-cell production [13].

There are different forms of probiotics preparations, and sometimes, their efficacy depends on whether they are single- or multi-strain preparations [14]. Compared to single-strain preparations, multi-strain probiotics contain more than one strain of the same species, genera, or multiple genera and sometimes including both bacteria and fungi (*Saccharomyces* species) [15]. Some single-strain probiotics are beneficial in alleviating gastrointestinal-tracts-associated diseases [16]. However, previous *in-vitro* studies showed that some multi-strain probiotics could exhibit better inhibitory effects on entero-pathogens, [17] and enhanced benefits by combining effects of different strains compared to their single-strain preparations [18]. Additionally, some multi-strain probiotics could reduce the absorption of harmful chemicals in humans and animals [19,20] due to their ability to absorb heavy metals within their cell walls [21]. Hence, prompting their application in biotechnology, detoxification therapy, and as dietary supplements [20,22]. The increased in the use of multi-strain probiotics has revealed optimal effects compared to single-strain probiotics [23]. However, despite the availability of multi-strain probiotics, not all had shown superior benefits [17], but overall, their effectiveness compared to single-strain probiotics are preferred [23]. Some multi-strain probiotics are more consistent in their actions than single-strain probiotics [15]. Therefore, this article discussed the mechanisms of synergy among constituent strains of multi-strain probiotics and their health benefits in humans and animals. However, very little data were found on the molecular mechanisms of cell-to-cell interactions among the isolates of multi-strain probiotics. Hence, the need for more robust and in-depth studies on this aspect.

## 2. Mechanisms of Action of Probiotics

**Figure 1:**
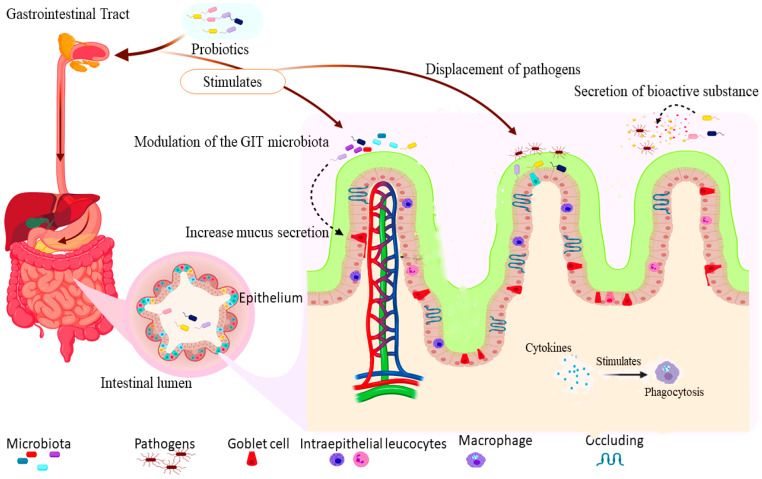
(created with BioRender; https://app.biorender.com/illustrations/edit/6001622bd73fad00a4e81c08, accessed on 28 November 2020) shows the mechanism of actions of probiotics: the intake of probiotics stimulates an increase in the secretion of mucus by goblet cells, mobilization of intraepithelial leucocytes, and tightening of the tight junctions to protect against the invasion of pathogens. The increase in mucus secretion and improvement of gut microbiota enhances competitive displacement and inhibition of pathogens adhesion to the gut epithelial surface. Furthermore, the action of bioactive substances such as lysozyme and cytokines stimulate phagocytosis by macrophages.

The mechanism of probiotics’ actions is the various means by which they exert their beneficial effects on the host, including immune modulation, stimulation/modulation of gut microbiota, stimulation of digestive enzymes, displacement of pathogens, and production of bioactive compounds [24,25,26]. The gut-associated actions are the principal effects of probiotics, also regarded as the basis of other health benefits [27] as summarized in Figure 1.

### 2.1. Stimulation of Bowel Microbiota

The gastrointestinal tract (GIT) is home to an organized microbial community (microbiota) which partake in metabolic, nutritional, biochemical, and immunological processes within the body. Hence, cell-to-cell interactions exist to regulate microbial multiplication, and preserve the intestinal homeostasis, leading to a range of host responses against commensal and pathogenic organisms [28]. The microbiota is an active ecosystem which is affected by many factors such as genetics, metabolism, nutrition, geographical location, stress, and antimicrobial treatment [29]. Some probiotics stimulate the action of the bowel microbiota [30], while others like *Bifidobacterium animalis* alter the microbiota’s metabolic pathways to increase the metabolism of carbohydrate and nucleotide while decreasing the metabolism of lipids and amino acids [31]. Similarly, yoghurt starter cultures containing *Streptococcus thermophilus* MK-10 and *Lactobacillus bulgaricus* 151 alter the gut microbiota in rats by increasing the population and diversity of mucosal microbes including enterobacteria, enterococci, and yeast [28]. These organisms benefit the host through maintenance and modification of favorable microbial population in the gut [14,32], and also restore the gut microbiota disrupted by antibiotic treatment [33] (Figure 1).

### 2.2. Immune Modulation

One of the essential mechanism of probiotics’ action is immune stimulation and immunomodulation against pathogenic microbes in the gastrointestinal tract (GIT) [34]. Probiotics influence the hosts’ immune system and have the ability to regulate inflammatory responses [35]. These effects were expressed in the intestine through the strengthening of the barrier protection resulting from the increased amounts of intra-epithelial leucocytes and goblet cells, and stimulation of the production of proinflammatory (tumor necrosis factor α (TNFα) and ILβ) and regulatory cytokines (TGFb, IL-10) for responses against pathogens, and the sustenance of mucosal integrity [36,37]. Multi-strain probiotics (containing *Lactococcus lactis* subspecies *lactis* CB460, *L. lactis* subspecies *cremoris* CB461, *Streptococcus thermopilus*, and *Propionibacterium freudenreichii* CB129) stimulates the production of anti-inflammatory cytokines; interleukin 10 and 12 (IL-10 and IL-12) in human peripheral blood mononuclear cells *in-vitro* [34]. Similarly, *Pediococcus acidilactici* and *Saccharomyces cerevisiae* subsp. *boulardii* mixture stimulate the production of proinflammatory cytokines (IL-6, IL-8, and TNFα) in the ileum of pigs experimentally infected with enterotoxigenic *Escherichia coli* (ETEC) F4 [38]. The lysozyme is an essential component of the innate immune system available in high quantity in the cytoplasmic granules of macrophages and polymorphonuclear neutrophils [39]. Neveling, et al. [40] reported an increase in the serum concentration of lysozyme which stimulates the activity of macrophages for phagocytosis by the administration of a multi-strain probiotic (containing *Lactobacillus crispatus, Lactobacillus salivarius, Lactobacillus gallinarum, Lactobacillus johnsonii, Enterococcus faecalis* and *Bifidobacterium amyloliquefaciens*) in chickens (Figure 1). The synthesis of interferon-γ (IFN-γ) by natural killer cells and Th1-lymphocytes stimulates production of oxidants with antimicrobial properties, also known to be upregulated by *Salmonellae* infections due to inflammatory responses [41]. However, the administration of a multi-strain probiotic (containing *Lactobacillus acidophilus, Lactobacillus fermentum, Lactobacillus plantarum*, and *Enterococcus faecium*) downregulates IFN-γ in chickens infected with *Salmonella enterica* [42]. Additional means by which probiotics modulate the immune system is through stimulation of mucus production and tightening of the intestinal tight junctions. Some strains of *Lactobacillus* and *Bifidobacterium* increase the expression of mucin on human intestinal cell lines and upregulate the tight junction protein (zona-occludens 1) which blocks pathogens from penetrating the *lamina propria* [27] (Figure 1).

### 2.3. Stimulation of the Digestive Enzymes

Some probiotics aid in the breakdown of complex macronutrients and provide the host with digestive enzymes and vitamins which enhance the absorption of nutrients [43,44]. Oak and Jha [27] showed the ability of some probiotics to lower lactose concentration in fermented food and increase the influx of lactase enzyme into the small intestine along with fermented food. The ability of probiotics to promote lactose fermentation has made them useful in treating lactose intolerance in humans [45]. Probiotics *Bifidobacterium* strains, such as *B. longum* and *B. animalis*, participate in the metabolism of oligosaccharides by secreting glycosyl hydrolases and stimulating beta-galactosidase activities [46]. Similarly, *Lactobacillus* species’ probiotic strains also exhibit high beta-galactosidase activities and increase insulin secretion, while *S. boulardii* expresses high disaccharidases alpha-glucosidases, alkaline phosphatases, and aminopeptidases activities [27].

Furthermore, feed supplementation with multi-strain probiotics (containing *Bacillus subtilis, Bacillus licheniformis* and *Lactobacillus strains*) revealed increased lipase activities and amylase enzymes within the gastrointestinal tract of shrimps [30]. Similarly, feeding of Nile tilapia (*Oreochromis niloticus*) with probiotics strains of *B. subtilis*, *L. rhamnosus*, and *S. cerevisiae* mixture also enhances amylase, lipase, and protease activities in the intestines [47]. The increase in digestive enzymes’ actions associated with some probiotics’ ingestion ultimately results in improved nutrients absorption, optimum feed utilization and growth performance [48]. In addition to the secretion of enzymes in the digestive tract, multi-strain probiotics containing *B. subtilis, E. faecium, L. reuteri*, and *P. acidilactici*, also enhances the intestinal surface area by increasing micro-villi density and heights, and immune integrity in tilapia fish [49]. The increase in villi heights was observed in heat-stressed broilers when fed with probiotics (containing strains of *B. subtilis*) [50]. Some probiotics were also found to stimulate the production of proteolytic and lipolytic enzymes helpful in the digestion of proteins and lipids, respectively [51].

### 2.4. Displacement of Possible Pathogens

One of the protective mechanisms of probiotics’ action is the competitive displacement of pathogens for adhesion and colonization of the mucosal surfaces [52] (Figure 1). Attachment to the intestinal mucosa is an essential factor associated with probiotics when there is intestinal inflammation [53]. Some probiotics microbes also share binding sites with some entero-pathogens and could hinder their attachment to the host’s cells by binding to the respective attachment sites [54]. Hence, justifying the rationale for using probiotics to protect against infections at early stages [52]. The adhesion of probiotics to the intestinal surface is necessary for the competitive displacement of pathogens and the modulation of immunological activities [55] (Figure 1). Additionally, the attachment of probiotics bacteria to the host’s mucosal surfaces increases the chance of host–probiotics interactions thereby resulting in temporary colonization and prolonging transit time within the intestine to cause their intended benefits [54].

### 2.5. Secretion of Bioactive Substances

The bioactive substances produced by lactic acid bacteria (LAB) include hydrogen peroxide, lactic acid, diacetyl, acetaldehyde, reuterin, and antimicrobial peptides [56] (Figure 1). For example, *B. subtilis* and *B. licheniformis* produce varieties of bioactive proteins, such as antibacterial peptides, chitinases, and dextranases which inhibits other pathogenic bacteria [57]. Antibacterial proteins were also isolated from LAB, among which bacteriocins constitute an essential group [58]. The bacteriocins are low-molecular-weight genetically encoded proteins synthesized in the ribosome and secreted outside the cell by some bacterial species [59]. These active peptides act by binding to surface receptors or invade host cells. Bacteriocins also act by pore formation on the target cells, cause cellular DNA degradation and inhibit the biosynthesis of peptidoglycan component of the bacterial cell wall [60]. The production of bacteriocin by LAB makes them inhibit pathogenic and food spoilage microbes, hence their potential for use as bio-preservatives [61]. Earlier research has revealed the presence of bacteriocin producing LAB strains from milk and cheese [62]. At a regulated pH (5.5), some produce gamma-aminobutyric acid (GABA) [63]; a neurotransmitter with antihypertensive activities [64]. Some probiotics can also stimulate the production of enzymes that hydrolyze bacterial toxins and modify toxin receptors in the host [65]. Different lactic acid bacteria species yield various bioactive substances that inhibit the proliferation of pathogenic microbes (Figure 1).

### 2.6. Mechanisms of Action of Multi-Strain Probiotics

Some multi-strain probiotics showed enhanced benefits due to the constituent strains’ synergy and additive effects resulting in high adhesion to the mucosae and pathogen inhibition within the digestive tract [66]. The genetics of the constituent species or strains of multi-microbial probiotics is vital for understanding the mechanisms by which they interact with each other, the intestinal microbiota, and the host. A comparative genomic analysis of the multi-strain probiotics VSL#3 by Douillard, et al. [67] revealed numerous genes that encode various bioactive substances associated with the probiotics’ health benefits. These include genes involved in lactose transport (*lacF*), peptidase activity, carbohydrate, metal, and amino acid transport. Some bacteria are endowed with extra-cellular structures known as fimbriae or pili that can adhere to the intestinal epithelium [68,69]. Two main types of pili are found in LAB and *Bifidobacteria*; the tight adherence pili (Tad pili) and the sortase-dependent pili where genes were found in some isolates of VSL#3 probiotics [67]. The Tad pilus gene clusters of *B. breve* (one of the VSL#3) strains are highly conserved and involved in gut colonization in mice [69]. All the isolates in VSL#3 also encoded cell-surface proteins carrying LPXTG motifs that enhance interactions with the host cells [67]. Several genes that encode fibronectin-binding region proteins, collagen adhesins, outer membrane proteins, fimbriae or pili were also identified in *L*. *helveticus* BD08, *L*. *plantarum* BP06, *L*. *acidophilus* BA05 and *B*. *animalis* subsp. *lactis* BL03 and BI04 with possible roles in host adherence [70]. *Lactobacillus plantarum* BP06 was shown to harbor the sortase-substrate, which were also present in *L. plantarum* WCFS1 in addition to a large mucus-binding protein that is O-glycosylated by N-acetyl-hexosamine [71]. These peptides are secreted in a glycosylated form and may have a host signaling function [72]. The presence of the genes encoding Tad pili, sortase-dependent pili, mucus binding proteins, some even glycosylated, and S-layer proteins involved in the interactions of probiotic isolates with one another, the host cells and the host’s microbiota showed that the combination of different species and strains might offer possible complementary, additive and synergistic effects in the gut [67]. Experimental assessment of the effects of single-strain probiotics of *L. helveticus* R0052, *B. longum* subsp. *infantis* R0033, and *B. bifidum* R0071 and the multi-strain preparation of all the three isolates revealed that the multi-strain synergistically influenced both T-helper type 1 (T_H_1) and T-helper type 2 (T_H_2) responses in Wistar rat models infected with enterotoxigenic *Escherichia coli* (ETEC) and *Nippostrongylus brasiliensis*, respectively [73]. The mechanism by which the multi-strain probiotics exerts their effects was proposed to be through the downregulation of the nuclear factor-kappa-B (NFκB) pathway [74].

The other mechanism by which bacteria communicate and regulate several genes’ expression is through a cell–cell communication known as quorum sensing (QS). Quorum sensing is based on the production, secretion, and detection of small signaling molecules, whose concentration correlates with the organisms’ cell density secreting these molecules in the surrounding [75]. An example of QS signaling molecules in bacteria is linked to autoinducer-2 (AI-2) which is synthesized through *Lux*S enzyme action [76]. Notably, Gram-positive bacteria use autoinducing peptides (AIP or peptide pheromones) that act as species-specific communication signals [75]. The AIP gene regularly borders a two-component regulatory system (QSTCS) gene cassette [77] which comprises the membrane located histidine protein kinase (HPK) that monitors environmental factors, and the cytoplasmic response regulator (RR) that modulates some specific genes expressions [77]. *L. plantarum* WCFS1 genome contains relatively high amount of peptide-based QS-TCS and other putative QS genes [78]. In the past, it was shown that interactions with other lactobacilli influenced the metabolic traits of *L. sanfranciscensis* and *L. plantarum* strains through *Lux*S-mediated mechanisms of QS [79].

### 2.7. Antagonisms among Multi-Strain Probiotics

Some probiotics and lactic acid bacterial strains produce antimicrobial substances ranging from organic acids to bacteriocins. Bacteriocins may be active against closely related strains, thereby suggesting the likelihood of antagonistic activities among closely related species or strains such as the lactic acid bacteria [66]. Previous studies have shown that the activation of some specific component regulatory systems such as plantaricin system regulated through the QS pathway by competing microorganisms could trigger microbial antagonism [80,81]. The growth of *L. sanfranciscensis* DPPMA174 and *P. pentosaceus* 2XA3 were shown to be inhibited when co-cultured with *L. plantarum* DC400 with an increased number of dead/damaged cells compared to their respective monocultures [80]. That was due to the biosynthesis of pheromone *Pln*A by *L. plantarum* DC400 either in a monoculture or co-culture conditions. The level of *Pln*A synthesis by *L. plantarum* DC400 is dependent on the co-cultivation microbe [82]. Hence, suggesting that co-culturing with *L. plantarum* DC400 might constitute stress to *L. sanfranciscensis* DPPMA174 and *P. pentosaceus* 2XA3. Another study by Di Cagno, et al. [75] showed that coculturing of *L. sanfranciscensis* CB1 and *L. brevis* CR13 or, *L. plantarum* DC400 constituted stress and inhibited *L. sanfranciscensis* CB1 with an increase in the number of dead/damaged cells and a decrease in the cultivable cell. This could have been due to several not easily definable conditions such as acid production, synthesis of antimicrobial compounds, ability to thrive in the medium, and competition for available nutrients.

## 3. Applications and Biological Functions of Multi-Strain Probiotics

Probiotics are live microorganisms with an expanded range of healthful activities, not just on the digestive tract but also on other body systems, including the urogenital and nervous systems [83]. There is increasing evidence in the biological applications of probiotics for the maintenance and improvement of gut health [34], inhibition of microbial pathogens and biofilms [84], improvement of human health through ingestion of fermented food products [85], and enhancement of growth and productivity in animals [86]. These and many other beneficial effects of probiotics made it necessary to review the different multi-strain probiotics applications, such as treating non-infectious diseases, inhibiting pathogens, and improving human and animal health.

### 3.1. Treatment of Diseases

Different randomized control clinical trials revealed that some specific probiotics are useful in the therapeutic management of gastrointestinal (GI) illnesses like inflammatory bowel disease (IBD) [34], irritable bowel syndrome (IBS), and pouchitis [87,88]. The administration of multi-strain probiotics containing different *Lactobacilli* species, *Streptococcus* and *Bifidobacterium* to patients who have systemic sclerosis alleviates the symptoms of gastrointestinal reflux and increased microbial alpha diversity group [88] (Table 1). Frech, et al. [89], suggested the intake of multi-strain probiotics in the amelioration of systemic sclerosis due to the association of the GI microbiome imbalance with the pathogenesis of the disease. Evaluating the functions of multi-strain probiotics containing a mixture of *Bifidobacterium, Lactobacillus*, and *Streptococcus* probiotics strains significantly alleviate the indicators of IBS including abdominal ache/distress and bloating and improved the compositions of intestinal microbiota in the treated patients [90]. Yoon, et al. [90] further proposed that these benefits were due to synergy between the different strains in the probiotic preparation since the action of probiotics is strain- and disease-specific.

Similarly, a multi-strain probiotic containing *S. boulardii, B. lactis, L. acidophilus, and L. plantarum* alleviated the signs of constipation, diarrhea, and modulates the microbial community in the small intestine of IBS patients [91]. The evidence of the existing relationship between alteration in the intestinal microbiome and cognitive behavioral changes is also increasing the application of probiotics [92]. In line with this, multi-strains probiotics consisting of *L. acidophilus*, *L. casei*, *B. bifidum*, and *L. fermentum* improve cognitive behavior in patients with Alzheimer’s disease [93]. Some multi-strain probiotics could significantly lower the level of circulating bacterial endotoxin in type-II diabetes mellitus patients [94]. Ingestion of multi-strain probiotics by pregnant women and their infants decreases food allergens’ sensitivity and the incidence of atopic eczema [95]. Consumption of yoghurt starter culture of *L. bulgaricus* 151 and *S. thermophilus* MK-10 mixture also relieves the symptoms of colitis in rats by increasing the colon length and the amount of mucosa-associated microbiota [28]. A decrease in the level of putrefactive short-chain fatty acid in the cecum contents of dextran sodium-sulphate salt-induced colitis in BALB/c rats was previously reported by Wasilewska, et al. [28]. A similar study also revealed the improvement of the same condition by a probiotic Dahi (made up of *L. acidophilus* LaVK2 and *B. bifidum* Bbvk3 mixture) through a reduction in myeloperoxidase action and level of TNF-α, IL-6, and IFN-γ in mice [96]. These studies showed the applications of multi-strain probiotics in alleviating inflammatory responses within the digestive tract, which might be useful in the treatment of different conditions, as shown in Table 1.

### 3.2. Inhibition of Pathogens

Recently, there is a rising concern over the high prevalence and spread of antimicrobial-resistant (AMR) pathogens worldwide. This global challenge is multifactorial and linked to selective pressure due to the frequent, prolonged, and irrational use of antibiotics in humans and animals [97]. The prolonged antimicrobial intake depletes the gut microbial populations thereby allowing the proliferation of pathogenic AMR pathogens like toxigenic *Clostridium difficile*, extended-spectrum β-lactamase (ESBL) producing *Enterobacteria*, methicillin-resistant *S. aureus* (MRSA), vancomycin-resistant *Enterococcus* species and other multi-drug resistant bacteria [98]. Patients on prolonged treatment with broad-spectrum antibiotics are at high risk of antibiotics-associated diarrhea and pseudomembranous colitis caused by antibiotic-resistant *C. difficile* and other pathogenic bacteria [99,100]. Some probiotics’ ability to modulate the intestinal microbiome is one of the mechanisms by which they prevent antibiotics-associated diarrhea and decrease the spread of AMR bacteria [101]. Lakhtin, et al. [102] reported that multi-strain probiotics consisting of *L. acidophilus* (strains NK1, K3III24, 100 ash), *Bifidobacterium adolescentis* MC 42, *B. bifidum*, and *B. gallinarum* GB synergistically produced lectins with antimicrobial activity against clinical strains of nystatin-resistant *Candida albicans*, *S. aureus*, and their biofilms. Experimental administration of probiotics to mice showed the inhibition of *C. difficile* by a multi-strain probiotic containing four strains of *E. faecalis* [103] (Table 1). Very similar work by Kondepudi, et al. [99] also showed the inhibition of *C. difficile* by the administration of multi-strain probiotics containing *L. plantarum* F44, *L. paracasei* F8, *B. breve* 46, and *B. lactis* to mice experimentally infected with *C. difficile*. The minimal side effects associated with the intake of probiotics for treatment coupled with the high incidence of reoccurrence of infections such as UTI [104] is also increasing the use of probiotics as a suitable alternative or adjunct therapeutic plan to conventional antimicrobials [33]. Probiotics do not leave residues or facilitate the development of resistance to antibiotics because they are preparations of live organisms [105].

Certain probiotics can modify or alter pathogenic microbial colonization in humans and animals [108]. Different multi-strain probiotics preparations significantly inhibit pathogenic bacteria such as *Vibrio cholerae* (in-vitro) [2], *S. aureus*, *S. epidermidis*, *Streptococcus pneumoniae*, *S.* *pyogenes*, *Propionibacterium acnes*, *Moraxella catarrhalis* [109], and *Proteus mirabilis* [84]. Similarly, cell-free supernatants of *Lactobacilli* inhibit non-Albicans *Candida* species biofilm formation and suggest its use for adjunctive treatment of oral candida infection [110]. These authors further suggested that some of these probiotic bacteria’s anti-biofilm activities are due to subtilisin and subtilin production, which are active antimicrobial molecules. Multi-strain probiotics containing a culture of *L. rhamnosus* and *L. reuteri* modify the vaginal microbial flora to decrease the vaginal coliforms and yeast in patients with bacterial vaginosis [104]. However, oral administration of multi-strain probiotics comprising *L. rhamnosus* GR-1 and *L. reuteri* RC-14 showed inactivity on the microbiome of the lower urinary system, rather an increase in the population of urinary pathogens was observed [108], thus, emphasizing the administration route as an essential factor for consideration to achieving the desired benefits.

A multi-strain probiotic containing different *Lactobacillus* strains hinders the adhesion of *E. coli* and *E. faecalis* to the bladder cell-lines, unlike the single-probiotics preparations [33] (Table 2). Likewise, the addition of feed-supplement fermented with a mixed culture of *S. cerevisiae*, *E. faecium*, *L. acidophilus*, and *B. subtilis* resulted in significant increase in serum immunoglobulin-M (IgM) level and inhibit the multiplication of *E. coli* in broilers [111]. Prophylactic and therapeutic administration of multi-strain probiotics of *L. acidophilus* (LA-5) and *B. animalis* subsp. *Lactis* (Bb12) mixture proved useful in preventing scar formation in the kidney of *E. coli*-induced pyelonephritis in a rat model [112]. Pre-treatment of chicks with certain multi-strain probiotics before exposure to pathogenic *S.* Enteritidis A9 inhibits the pathogens from colonizing the birds [40] (Table 2). Similarly, the administration of multi-strain probiotics and a recombinant attenuated *Salmonella* vaccine confers protection against avian pathogenic *E. coli* and *Salmonella* Kentucky in White Leghorn chicks [113]. The *in-vitro* study of a multi-strain probiotic containing *L. plantarum* (strain L21 and strain L80) and *L. paraplantarum* (strain L103) revealed the inhibition of *E. coli*, *Salmonella* groups B and D in a co-culture tested using agar-spot assay [114]. Multi-strain probiotics mixture of *L. casei* and *E. faecium* also showed significant inhibition of *Entamoeba invadens* (the causative agent of traveler’s diarrhea in humans) [115].

### 3.3. Improvement of Human Health

The health effects of probiotics are necessitating its commercial development due to their worldwide consumption [85]. The benefits accrued to ingestion of fermented milk containing probiotic bacteria include decreased total cholesterol, enhanced immunity by facilitating resistance to infection and bacterial inhibition and preventing oxidative stress during exhaustive bodily activities [119]. Ingestion of probiotics is known to improve gut health in humans and animals [120]. In humans, probiotics have beneficial effects on the nervous [93], gastrointestinal [121], and immune systems [122]. Some probiotics alleviate various gastrointestinal ailments like irritable bowel disease (IBD) and systemic sclerosis in humans [89]. Multi-strain probiotics had proved beneficial for the treatment of dysentery in addition to the standard regimen with a marked reduction in the extent of bloody stooling, and a decreased average length of hospital stay [123]. These effects were a result of the alteration of the microbial and metabolic activities within the gut, and which are enough to modify the disease process and pathological conditions [124].

### 3.4. Multi-Strains Probiotics in Animal Husbandry

The increase in the demand for animal proteins due to the global population’s rise is overstretching the livestock industry, leading to the use of antibiotics as growth promoters and prophylaxis against infectious agents [125]. However, this strategy is not sustainable because of the growing concerns about antibiotic resistance among microbial pathogens [126]. Hence, resulting in restrictions on the non-therapeutic administration of antibiotics to animals [125] in different regions of the world. Therefore, probiotics may be a potential alternative for improving gastrointestinal health and growth promotion in different animal species [86]. Based on these, the roles of probiotics in the various livestock sub-sectors, including poultry, aquaculture, piggery, and ruminant nutrition were discussed as follows.

### 3.5. Poultry Farming

In poultry, the addition of probiotics derived from *Lactobacillus, Bacillus* [127], and *Clostridium* species to feed has a positive impact on the growth yield, feed digestion [128], immunity [129], meat quality [130], and coliforms bacterial count [86,131]. The administration of multi-strain probiotics (comprising of *L. acidophilus* LAP5, *L. fermentum* P2, *P. acidophilus* LS, and *L. casei* L21) to specific-pathogen-free (SPF) chicks infected with *Salmonella enterica* subspecies *enterica* decreases the abundance of proteobacteria of which Salmonella is a member [9]. Likewise, multi-strain probiotics containing *L. acidophilus*, *B. subtilis* DSM 17299, and *Clostridium butyricum* increases the serum level of IgA and IgM with a decline in the count of *E. coli* in the feces of broiler chickens [86]. Furthermore, supplementing poultry feed with multi-strain probiotics was reported to result in a rise in villus length and the number of goblet cells in the jejunum, and villus height to crypt ratio in the ileum of chickens [132]. Feeding of chickens with citrus-Junos by-product fermented with multi-strain probiotics (containing *S. cerevisiae*, *E. faecium*, *L. acidophilus* and *B. subtilis*) also increases the weight gain and mean daily feed consumption [111]. Multi-strain probiotics of *B. subtilis* (*B. subtilis 1781* plus *B. subtilis* 747 or *B. subtilis* 1104 plus *B. subtilis* 747) boost the bowel immunity and strengthen the veracity of the gut barrier by stiffening the gut tight junctions in chickens [133]. In laying chickens, administration of multi-strain probiotics reduces feed conversion ratio and percentage of damaged eggs [134]. These studies are advocating probiotics to chickens feed to promote growth performance and health through enhancement of digestive function and regulation of intestinal microbiome [129,135]. However, several works have also reported a little or no improvement in the total increase of weight in chickens administered with probiotic (including multi-strain) supplemented feed [136]. These differences could be a result of variations in the strains or species used in the formulation of the probiotics, preparation techniques, the dosage administered, age of the birds and general level of sanitation [86,128].

### 3.6. Aquaculture

The use of probiotics for health improvement has also found application in aquaculture. The addition of multi-strain probiotics in the feed of rohu (*Labeo rohita*) was revealed to stimulate cellulolytic and amylolytic enzymes secretions with improved the growth output [137]. The multi-strain culture of *B. subtilis*, *B. licheniformis*, and *lactobacilli* probiotics significantly improves pacific white shrimps’ growth (*Litopenaeus vannamei*) and enhances non-specific immunity and the abundance of *Bacillus* to influence the intestinal microbiota [30]. Similarly, a cocktail of *Lactobacillus pentosus* BD6, *L. fermentum* LW2, *B. subtilis* E20, and *S. cerevisiae* P13 probiotics enhanced the health and growth output of white shrimp (*L. vannamei*) compared to single-strain probiotics [6]. These works positioned that the improved growth noted from the administration of the multi-strain probiotic is due to a synergy or additive effects observed by the individual strains in the preparation resulting in enhanced enzyme activity during digestion and better feed conversion [138]. Multi-strain probiotics (containing *B. subtilis, E. faecium, L. reuteri*, and *P. acidilactici*) also results in improved growth yield, and bowel immunity through the elevation of proinflammatory cytokines (TNFα and ILβ) in the intestines of tilapia fish [139]. Unlike the previously cited works, multi-strain probiotics decrease the growth rate in mud-crabs when fed with *B. subtilis* E20 and *L. plantarum* 7–40 combination supplemented feed due to antagonism [140]. These studies’ findings imply a careful selection of strains for inclusion in multi-strain preparations [6].

### 3.7. Swine Production/Piggery

The weaning period in piggery coupled with diets changes from simply digestible (milk) to solid feeds may result in intestinal perturbation, thereby causing diarrhea and slow growth rate [141,142,143]. The stress of post-weaning alongside the alteration in the gut microbiota results in post-weaning diarrhea; a severe health challenge characterized by diarrhea, death in severe cases, and substantial monetary implications in piggery [38]. Post-weaning diarrhea may result from the rapid multiplication of enterotoxigenic *E. coli* (ETEC) and primarily affects piglets two weeks after weaning [144]. The treatment of this economic disease is by using antibiotics; however, the current surge of AMR bacteria had necessitated the demand for farmers to look for an alternative treatment to prevent and control diseases in their livestock [38]. Probiotics may serve as viable options to antibiotics in piggery, especially for non-therapeutic usage and growth enhancement [145]. The ingestion of probiotic bacteria (like *P. acidilactici*) and yeast (*S. cerevisiae boulardii*) protect from microbial infection by enhancing intestinal defenses and performance in different monogastric animals [146]. In line with this, piglets fed with multi-strain probiotics consisting of *L. reuteri* (strains VB4 and ZJ625), *Streptococcus salivarius* NBCR 13956, and *L. salivarius* ZJ614 have better mean regular weight increase and feed utilization, unlike the single-strain and control groups [141].

Furthermore, the administration of some certain multi-strain probiotics to pigs results in weight gain [125] and inhibits the attachment of Enterotoxigenic *E. coli* (ETEC) F4 to ileal mucosa of piglets [38]. This finding was supported by the discoveries of the modulatory effects of *P. acidilactici* and *S. cerevisiae boulardii* either as mixed or single preparations on establishing microbial population such as members of the family Bifidobacteriaceae and Lactobacillaceae in the porcine bowel [147]. These are therefore advised for a careful screening and inclusion of probiotics microbes with proven properties to play vital roles in the improvement of growths of piglets, primarily through the post-weaning period. Similarly, multi-strain probiotics stimulate a rise in the level of proinflammatory cytokines (TNF-α, IL-6, IL-4, IL-10, and TGF-β) and antibodies in the colostrum of sows when administered during pregnancy and lactation, hence offering protection to both the dam and neonates through stimulation of cellular immunity [148].

### 3.8. Ruminants Nutrition and Production

Some probiotics are suitable supplements in livestock feeds and may improve the rumen’s microbial ecosystem, enhance feed digestion, and restores gut microflora in diarrhea in ruminants [149]. The administration of lactobacilli probiotics enhances calves’ overall health status [150]. Consequently, a probiotic mix (containing *E. faecium*, *B. bifidum, P. acidilactici, L. acidophilus*, *L. casei*, peptide extract, an enzyme blend and killed yeast extract) significantly shortens the duration of diarrhea in dairy calves at the onset of diarrhea [151]. Additionally, several studies reported using probiotics to control diarrhea, improve average daily weight gain, and feed efficiency in calves [151,152,153]. The administration of multi-strain probiotics (made up of *Lactobacillus sakei* FUA3089 and *P. acidilactici* FUA3138 and FUA3140) modulates specific serum metabolites, milk components, and increased milk production in dairy cows [154]. Similarly, feeding of dairy cows with pasture from paddocks treated with multi-strain probiotics (containing *L. parafarraginis*, *L. buchneri*, *L. rapi*, *L. zeae*, *Acetobacter fabarum* and *Candida ethanolica*) showed a higher volume of milk and protein content in the treatment group compared to the control group [155].

### 3.9. Synbiosis of Multi-Strain Probiotics with Other Biologically Active Molecules

Some probiotics are prepared as synbiotics (prebiotics) along with other active substances for maximum physiological effects. Ingestion of synbiotics made of multi-strain probiotics (containing *L. acidophilus* strain T16, *L. casei* strain T2 and *B. bifidum* strain T1) and 800 mg inulin (HPX) by gravid women with gestational diabetes mellitus decrease the rate of caesarean section and hyperbilirubinemia and hospitalization of newborns [156]. Administration of synbiotics (containing multi-strain probiotics and prebiotics) may alleviate some digestive system conditions, sepsis, and death in preterm babies [157] (Table 3). Treatment of vaginal candidiasis in patients is enhanced by administering azoles alongside multi-strain probiotics comprising *L. acidophilus*, *L. rhamnosus*, *S. thermophilus*, and *L. delbrueckii* subsp. *Bulgaricus* [158] (Table 3). This study further reiterates the potentials of multi-strain probiotics combination for treatment and deterrence of recurrence of vaginal candidiasis, especially in cases of azole-resistant mycosis. In poultry, co-administration of a specific multi-strain probiotic mixture and zinc to broiler increases the final body weight, feed efficiency, total goblet cells, and ileal villus height unlike in the control group [159]. Similarly, short-term administration of multi-strain probiotic-synbiotics to laying chickens infected with *Salmonella typhimurium* (*S. typhimurium*) positively modulated the caecal microbiota but had no marked effect on shedding of *S. typhimurium* [65] (Table 3).

## 4. Conclusion and Future Consideration

Several studies suggest using multi-strains probiotics to prevent and treat different kinds of conditions from non-infectious to infectious diseases. Most of these studies emphasized probiotics in diets and have shown several derived benefits from such administrations. The potential of individual probiotic organisms to act in synergy or additively when in a mixture, holds a grand promise for future use in treating various diseases. Based on this review, probiotic organisms could secrete various substances that can inhibit the multiplication of pathogenic microbes, which is vital for future considerations because of the safety in its consumption and the associated health benefits. The current global challenges associated with the rise and spread of antimicrobial resistance by several pathogens had provoked the need for suitable alternatives to the current antibiotics, thus, indicating the need for further development of probiotics because of its potential in producing several bioactive compounds like lectins, bacteriocins, bioactive proteins, and antibacterial peptides that are inhibitory to pathogenic antibiotic-resistant bacteria and some fungi. The production of these essential bioactive peptides could be harnessed for further development into bio-additives to be used instead of the whole-cell probiotics formulations. In animals, feed supplementation with probiotics has proven helpful by improving growth performance and weight gain, meat quality, and humoral immunity and decreasing pathogenic microbes’ shedding. The use of probiotics could gradually outshine the prophylactic applications of antimicrobial drugs for growth enhancement in animals, thereby decreasing the spread of antimicrobial-resistant pathogens. Furthermore, this review had also shown that probiotics could be used in combination with prebiotics as symbiotics to give maximum benefits for better physiological effects. Therapeutic use of probiotics in a mixture with other compounds such as zinc and some antimycotic agents could exert more effects compared to the usage of those substances alone.

Finally, to maximize all the benefits associated with probiotics consumption, research should determine the specific mechanisms of actions of probiotics microbes for more specific applications in respective disease conditions. There is also the need to study and understand each probiotics strain’s best combination because some bacteria act synergistically, some additively, and some antagonistically. Additionally, the bioactive substances produced by some probiotics could be extracted to formulate supplements for use in specific conditions where individuals showed some reactions to the consumption of the whole-cells preparations. The harvesting and harnessing of the bioactive substances produced by individual constituents of mixed probiotics could also solve the challenges associated with the inconsistency of viable cells when live microbes are used. That will also enable large-scale production for commercialization. Finally, further studies in this direction could be an essential factor in the future research and development of multi-strain probiotics. 

## Figures and Tables

**Table 1:** The use of multi-strain probiotics in disease treatment.

Probiotics Mixture	Conditions	Mechanism of Actions	References
*B. bifidum* W23, *B. lactis* W52, *L. acidophilus* W37, *L. brevis* W63, *L. casei* W56, *L. salivarius* W24, *Lactococcus lactis* W19 and *L. lactis* W58	Endotoxins	Improvement of endothelial barrier, inhibition of mast cell, activation of proinflammatory cytokines, and decrease endotoxin	[94]
*L. acidophilus*, *L. casei*, *B. bifidum*, and *L. fermentum*	Cognitive function in Alzheimer’s disease		[93]
*L. paracasei* DSM 24,733, *L. plantarum* DSM 24,730, *L. acidophilus* DSM 24,735, and *L. delbrueckii* subspecies *bulgaricus* DSM 24,734), Bifidobacteria (*B. longum* DSM 24,736, *B. breve* DSM 24,732, and *B. infantis* DSM 24,737), and Streptococcus (*S. thermophilus* DSM 24,731)	Systemic sclerosis-associated gastrointestinal disease	Improvement of GI reflux and intestinal microbiota alpha diversity	[89]
*L. acidophilus* LaVK2 and *B. bifidum* Bbvk3	Dextran sodium- sulphate salt-induced ulcerative colitis in mice	Reduction in myeloperoxidase activity, levels of TNF-α, IL-6, and IFN-γ	[96]
*L. bulgaricus* 151 and *S. thermophilus* MK-10	Dextran sodium- sulphate salt-induced colitis	Modulation of intestinal microbiota, decrease the content of putrefactive short-chain fatty acid, enhanced production of cytokines	[28]
*B. bifidum* (KCTC 12199BP), *B. lactis* (KCTC 11904BP), *B. longum* (KCTC 12200BP), *L. acidophilus* (KCTC 11906BP), *L. rhamnosus* (KCTC 12202BP) and *S. thermophilus* (KCTC 11870BP)	Irritable Bowel Syndrome (IBS)	Alleviation of IBS symptoms and improvement of intestinal microbiota	[90]
*B. longum* and *L. casei* strain Shirota	Treatment of obesity	Decreased weight and triglyceride in rats fed with the high-fat diet.	[106]
*S. boulardii*, *L. acidophilus*, *L. plantarum*, *B. lactis*	IBS associated with bacterial overgrowth and constipation	Improvement in bloating, and pain associated with constipation	[91]
*L. plantarum*, *B. breve*, and *L. fermentum*	high-dietary fat-induced obesity and *E. coli* challenged	Causes reduced Lipopolysaccharide and IL-1β, improved the structure of intestinal flora and increased the fecal short-chain fatty acid (SCFA) content	[107]

**Table 2:** Multi-strains probiotics against pathogenic microbes.

Multi-Strain Probiotics Isolates	Pathogenic Bacteria	Host	References
*B. subtilis* and *L. mesentroides*	*Vibrio cholereae*	In-vitro agar diffusion test	[2]
*L. plantarum* F44, *L. paracasei* F8, *B. breve* 46 and *B. lactis*	*Clostridium difficile*	Mice	[99]
*S. oralis* and *S. salivarius*	Biofilm (*S. aureus*, *S. epidermidis*, *S. pneumoniae*, *S. pyogenes*, *Propionibacterium acnes* and *Moraxella catarrhalis*	Dogs	[109]
*L. acidophilus* LAP5, *L. fermentum* P2, *P. acidophilus* LS, and *L. casei* L21	*S. enterica* subspecies *Enterica*	Chickens	[9]
*L. acidophilus* LA-5 and *B. bifidum* BB-12	*P. stomatis*, *P. multocida*, *P. canis*, *N. animaloris*, and *N. zoodegmatis*		[116]
*P. acidilactici* and *S. cerevisiae boulardii*	Enterotoxigenic *E. coli* (ETEC) F4	Pigs	[38]
*L. acidophilus* NCIMB 30184, *L. fermentum* NCIMB 30226, *L. plantarum* NCIMB 30187, and *L. rhamnosus* NCIMB 30188	Pathogenic *E. coli* and *E. faecalis*		[33]
*S. cerevisiae*, *E. faecium*, *L. acidophilus* and *Bacillus subtilis*	*E. coli*	Chickens (broilers)	[117]
*L. acidophilus* NCIMB 30184, *L. rhamnosus* NCIMB 30188, *L. plantarum* NCIMB 30187, *L. delbrueckii* ssp. bulgaricus NCIMB 30186, *L. casei* NCIMB 30185, *L. lactis* NCIMB 30222, *L. salivarius* NCIMB 30225, *L. fermentum* NCIMB 30226, *L. helveticus* NCIMB 30224, *B. bifidum* NCIMB 30179, *B. breve* NCIMB 30180, *B. infantis* NCIMB 30181, *B. longum* NCIMB 30182, *S.* thermophilus NCIMB 30189 *B. subtilis* NCIMB 30223	*S. typhimurium*, *C. difficile*	In-vitro distal colon model	[118]
*L. acidophilus* NCIMB 30184, *L. fermentum* NCIMB 30188, *L. plantarum* NCIMB 30187 and *L. rhamnosus* NCIMB 30226	*E. faecalis* NCTC 0075 and *E. coli* NCTC 9001	In-vitro agar diffusion test	[17]
*L. rhamnosus* and *L. reuteri*	Vaginal coliforms and yeast	Human (female)	[104]
*L. crispatus*, *L. salivarius*, *L. gallinarum*, *L. johnsonii*, *E. faecalis* and *B. amyloliquefaciens*	*Salmonella* Enteritidis A9	Chickens (broiler)	[40]
*L. acidophilus, L. fermentum*, *L. plantarum* and *E. faecium*	*Salmonella enterica*	Chickens (broiler)	[42]
*B. amyloliquefaciens* B-1895 and *B. subtilis* KATMIRA1933	Inhibits *Proteus mirabilis* biofilm formation	Invitro	[84]
*E. faecalis* (strains NM815, and NM915) and *E. faecium* NM1015	*C. difficile* infection	Mice	[103]
*L. acidophilus* (LA-5), and *B. animalis* subspecies *Lactis* (Bb12)	*E. coli* induced pyelonephritis	Sprague-Dawley rat	[112]
*L. casei* and *E. faecium*	*Entamoeba invadens*	Invitro	[115]
*B. subtilis, L. acidophilus*, *P. acidilactici*, *P. pentosus*, *Saccharomyces pastorianus*	Avian pathogenic *E. coli* and *Salmonella* Kentucky	White leg-horn chicks	[113]
*L. gasseri* and *L. rhamnosus*	Non-*Candida albicans* biofilm formation	In-vitro	[110]

**Table 3:** Use of multi-strain probiotics along with other substances.

Synbiotics	Actions	Host	References
*L. acidophilus* strain T16, *L. casei* strain T2) and *B. bifidum* strain T1 plus 800mg inulin (HPX)	decreased the incidence of cesarean section rate and newborn’s hyperbilirubinemia and hospitalization	Human (pregnant women)	[156]
*L. acidophilus*, *L. rhamnosus*, *S. thermophilus*, and *L. delbrueckii* subspecies *Bulgaricus* plus fluconazole	Enhance the treatment of Vaginal candidiasis caused *Candida albicans*	humans	[158]
*L. plantarum, L. acidophilus, L. delbrueckii subspecies bulgaricus, B. bifidum, L. rhamnosus, E. faecium, S. salivarius subspecies thermophilus, Aspergillus oryza*, and Candida *pintolopesii* plus Zinc	Enhances growth performance, better feed utilization, increase in villus height in the duodenum and ileum	Chicken (broiler)	[159]
Synbiotics A: *Enterococcus* sp., *Pediococcus* sp., *Bifidobacterium* sp., *Lactobacillus* sp. plus fructooligosaccharides Synbiotic B: *L. acidophilus, L. casei, L. salivarius, L. plantarum, L. rhamnosus, L. brevis, B. bifidum, B. lactis, S. thermophilus*, prebiotic inulin (chicory root extract), protease, amylase, cellulase, hemicellulase, lipase, papain and bromelain	Modulate the caecal microbiota without any effects on Salmonella *Typhimurium* shedding	Chickens (layers)	[65]
Probiotics; (*L. rhamnosus, L. casei L. plantarum B. animalis*) prebiotics (383 mg of fructooligosaccharides and 100 mg of galactooligosaccharides)	Improved gastrointestinal complications, sepsis, and mortality in premature infants	Preterm infants	[157]

## Data Availability

Not applicable.
